# Small protein sequences can induce cellular uptake of complex nanohybrids

**DOI:** 10.3762/bjnano.10.238

**Published:** 2019-12-12

**Authors:** Jan-Philip Merkl, Malak Safi, Christian Schmidtke, Fadi Aldeek, Johannes Ostermann, Tatiana Domitrovic, Sebastian Gärtner, John E Johnson, Horst Weller, Hedi Mattoussi

**Affiliations:** 1 Department of Chemistry and Biochemistry, Florida State University, 95 Chieftan Way, Tallahassee, Florida 32306, United States; 2 Institute of Physical Chemistry; University of Hamburg, Grindelallee 117, 20146 Hamburg, Germany; 3 The Hamburg Center for Ultrafast Imaging, University of Hamburg, Luruper Chaussee 149, 22761 Hamburg, Germany; 4 Laboratoire Physique des Solides, UMR 8502, Université de Paris Sud bât 510, 91405 Orsay Cedex, France; 5 Altria Center for Research and Technology, 601 E Jackson Street, Richmond, VA, 23219, United States; 6 Center for Applied Nanotechnology (CAN) GmbH, Grindelallee 117, 20146 Hamburg, Germany; 7 Instituto de Microbiologia Paulo de Goes, Universidade Federal do Rio de Janeiro, 310. Lab I014, 21941-902, Rio de Janeiro, Brazil; 8 The Scripps Research Institute, Department of Integrative Structural and Computational Biology, MB31, La Jolla, California 92037, United States; 9 Universitätsklinikum Hamburg Eppendorf, 20246, Martinistraße 52, 20251 Hamburg, Germany

**Keywords:** bioconjugation, cellular uptake, nanoparticle hybrids, polymer encapsulation, self-assembly

## Abstract

In this letter, we report on the ability of functional fusion proteins presenting a lytic gamma peptide, to promote interactions with HeLa cells and delivery of large hybrid nanostructures.

## Introduction

Developing hybrid nanostructures made of more than one component nanomaterial, combined with biomolecules is a highly sought goal in biomedical science, and can find applications in multimodal imaging and therapeutics [1–2]. Although interest in developing such hybrid nanostructures by, for example, combining plasmonic and fluorescent, or magnetic and fluorescent nanoparticles have attracted much attention for the development of bioassays, their use as cellular labelling platforms has been less explored [2–3]. A few demonstrations describing the use of such hybrid nanostructures in cell labelling have been recently reported. In one study, Jana and co-workers reported the design of fluorescent and plasmonic nanohybrids by covalent attachment of luminescent quantum dots (QDs) and Au nanorods. Further functionalization with glucose, using glutaraldehyde coupling chemistry, yielded nanohybrids that could subsequently be used for the staining of cell membranes [4]. In two separate studies, Chan and co-workers described two interesting hybrid systems. In the first, a charge driven self-assembly of AuNPs and different-colour QDs into multicolour, non-blinking nanohybrids was introduced. These nanohybrids were then coupled to various proteins, and among them the human transferrin protein was found to induce the highest intracellular uptake following 24 h incubation of these hybrids with cell cultures [5]. In the second, functional colloidal superstructures assembled using DNA linkers elicited a reduction in the response of macrophages to these hybrid materials combined with an improvement in their in vivo tumour accumulation [6]. Weil and co-workers described the use of multimodal platforms, made of diamond dots combined with gold nanoparticles, as imaging probes of live cell cultures [7]. We have recently characterized a hybrid system consisting of self-assembled gold nanoparticles (AuNPs) and polymer-encapsulated QDs. These constructs were further functionalized with polyhistidine-tagged proteins, yielding functional conjugates that exhibit fluorescent and plasmonic properties [8].

Over the last two decades several groups have investigated mechanisms for intracellular-uptake and in vivo biodistribution of various nanomaterials [9–11]. Due to the complexity of nanostructured materials combined with the intricacy of cell biology, it has been proven very difficult to develop a good understanding of what controls the processes involved in the intracellular uptake and ensuing distribution of various nanomaterials [9]. Several studies have consistently found that NPs are very often taken up by endocytosis, and once inside the cells they remain trapped within endosomal compartments [10,12]. A few other studies reported that a sizable fraction of the delivered nanoparticles can end up in the cytoplasm, by either circumventing endocytosis through the use of virus-derived peptide sequences, or non-disruptively penetrating the cellular membranes [13]. Escape from endosomal vesicles of once endocytosed nanoparticles have also been discussed [12,14–15].

More recently, there have been a few reports discussing the use of luminescent Eu-loaded hydroxyapatite nanocrystals for rapid HeLa cancer cell imaging [9,11,16], or the nanostructure self‐assembly driven by amino acid coordination to increase the biological stability and tumour accumulation of curcumin [17]. Overall, there is a consensus that using colloidally stable nanoparticles is crucial for understanding and controlling cellular uptake, because materials that are prone to aggregation show higher non-specific interactions with biological fluids and cell membranes [18–19].

Here, we report on the use of a lytic gamma peptide (γ-peptide) derived from the *Nudaurelia Capensis Omega* virus (NωV), which was genetically fused onto maltose binding protein appended with 6-histidine tag, (His_6_-MBP-γ), to promote the intracellular delivery of hybrid QD-AuNP assemblies [20–21]. This peptide is produced during viral capsid maturation and is thought to enable cellular internalization of the virus. It has been shown that the MBP-fused γ-peptide is able to disrupt artificial liposomes [20–21]. Recently, we have used this His_6_-MBP-γ to promote the uptake of QDs by mammalian cells [22]. Here, we expand this approach to test the peptide capacity to promote the intracellular uptake of more complex hybrid nanostructures made of self-assembled QDs and AuNPs.

## Results and Discussion

**Figure 1:**
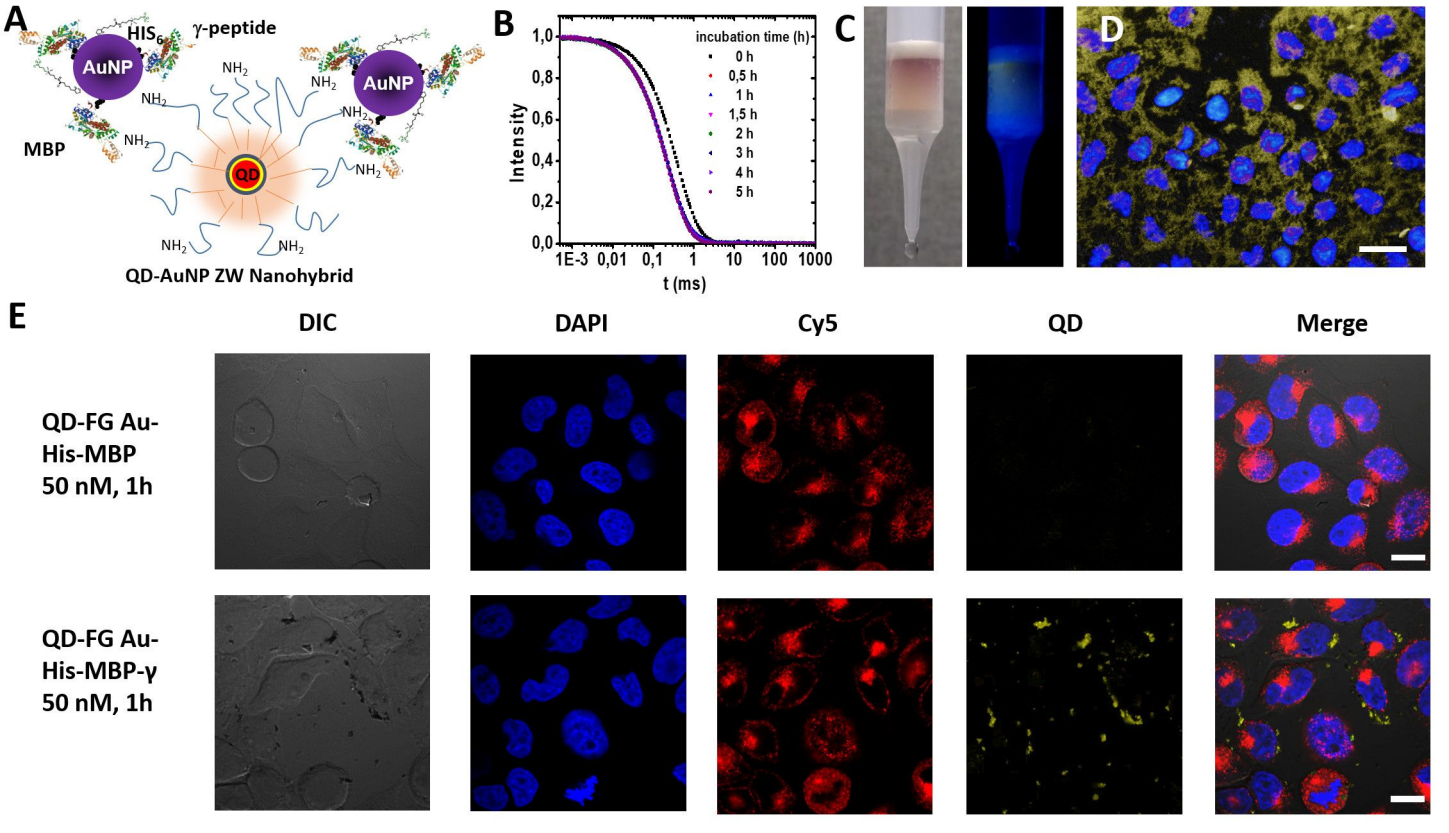
(A) Schematic representation of the nanohybrids (not to scale). The central QD (red, yellow, blue = core/shell/shell) is embedded in a crosslinked polymer micelle, consisting of a hydrophobic block (red) and an amphiphilic block (PEO). Interaction of the QDs with AuNPs (purple) is driven by the amine functional group. Gold nanoparticles are His-conjugated to His_6_-MBP-γ. The entire nanohybrid has a hydrodynamic diameter of ≈90 nm. (B) Profiles of the intensity autocorrelation function, collected from a dispersion of the nanohybrids in Dulbecco's Modified Eagle Medium (DMEM) media, at various time intervals over a period of 5 h. The profiles are unchanged, indicating colloidal stability. (C) Binding of the nanohybrids (presenting MBP-γ) onto an amylose column; the fluorescent and pinkish band reflect the presence of QDs and AuNPs. (D) A representative epifluorescence image of fixed HeLa cells after incubation with the nanohybrids at *c*(QD) = 100 nM and 14 His_6_-MBP-γ equiv/AuNP, scale bar = 50 µm. (E) Confocal microscopy images of HeLa cells incubated with nanohybrids for 1 h; *c*(QD) = 50 nM and 7 His_6_-MBP-γ equiv/AuNP. 60× magnification was used. Scale bar = 20 µm. (Top panels) data correspond to nanohybrids containing His_6_-MBP (no γ-peptide); (bottom panels) correspond to nanohybrids with His_6_-MBP-γ. Shown are differential interference contrast (DIC), 4′,6-Diamidino-2-phenylindol (DAPI), Cy5-red, and yellow QD channels, along with the merged images. Figure 1A adapted with permission from [8], copyright 2016 American Chemical Society.

The biologically active plasmonic–fluorescent hybrids were formed using a self-assembly route which relies on direct metal-coordination interactions. Here, amine-to-gold and imidazole-to-gold coordination were applied to couple QDs and AuNPs, or to conjugate His_6_-MBP-γ onto the AuNPs, respectively [8,23]. AuNPs stabilized with zwitterion-modified lipoic acid (LA-ZW-AuNPs) were selected for this study, due to their compact size, enhanced colloidal stability, and reduced non-specific interactions in biological media [22,24–31]. The central QDs used to build up the hybrid assemblies were prepared via encapsulation within a polymer coating made of an amine-functionalized polyisoprene-*block*-polyethylene oxide (PI-*b*-PEO-NH_2_). The lateral amine groups allowed attachments to AuNPs, which then served for the immobilization of a few His_6_-MBP-γ, as schematically shown in Figure 1A.

To demonstrate the potential utility of this nanohybrid system in biology, colloidal stability studies in culture media were first carried out. We found no sign of aggregation build up for at least 5 hours, as verified using dynamic light scattering measurements, where mono-modal autocorrelation function along with a single intensity vs hydrodynamic size peak (i.e., Laplace transform profile) were acquired (see Figure 1B) [17]. The hydrodynamic diameter of the entire nanohybrid is approx. 90 nm; this is larger than the values measured for LA-ZW-AuNPs (*D*_H_ ≈ 10 nm) and for QD-NH_2_ (*D*_H_ ≈ 30 nm) and the dimension of the MBP (an ellipsoidal with overall dimensions of ≈3 × 4 × 6.5 nm) [8,32]. In a control experiment, using citrate-stabilized AuNPs in a similar assay, the hybrid self-assembly precipitated within few minutes. This behaviour is attributed to the nature of the citrate coating (weak stabilizer), and further proves that using LA-ZW-AuNPs enhances their colloidal stability of the whole assembly, yielding a platform suitable for investigating interactions with cells. Further details on the stability under additional conditions are provided in Supporting Information File 1.

We first tested the biological activity of the His_6_-MBP-γ in the hybrids, as done in reference [30]. We found that once uploaded onto an amylose-filled column, the nanohybrid stayed tightly bound to the column even after several washes with buffer. The bimodal character of the hybrid is reflected in the pinkish colour of the AuNPs and the fluorescence of the QDs of the immobilized band in the amylose column (see Figure 1C). The band could be readily released by adding a few mL (10–20) of maltose solution. This release is promoted by the stronger affinity of maltose (the substrate for MBP) to the bound His_6_-MBP-γ. Overall, this experiment clearly proves that the nanohybrids contain MBP and that the bound MBP stays functional [8,23,28]. Further details are available in Supporting Information File 1.

After confirming the structural integrity and colloidal stability of the nanohybrids, we then proceeded to probe their interactions with HeLa cell cultures. For this, dispersions made of consisting of 100 nM QD solution, 2 equivalents of LA-ZW-AuNP per QD and 14 equivalents His_6_-MBP-γ per AuNPs, were incubated with the cell culture for 1 h. Following rinsing the culture was imaged using epifluorescence and confocal fluorescence microscopy. A pronounced intracellular uptake of the hybrids was observed, as indicated by the significant fluorescence staining of the cells (see Figure 1D). Additional confocal images collected from two sets of cultures, one incubated with nanohybrids prepared with His_6_-MBP-γ and the other with His_6_-MBP (gamma-free MBP), and serving as control. Only the culture incubated with nanohybrids prepared with His_6_-MBP-γ yielded pronounced intracellular staining; the control cultures did not show any cellular uptake (see Figure 1D and Figures S3 and S4 in Supporting Information File 1). In addition, the distribution of the QD staining (shown in Figure 1E, top panels) is not fully overlapped with the endosomal compartments counterstained with a red dye.

We tested the effects of decreasing the overall concentration of the nanohybrids or the number of MBP-γ per nanohybrid assembly on the staining levels of the cells. We found that reducing the overall concentration of the overall hybrids, the QD-to-AuNP molar ratio in the hybrids, the incubation time to 30 min, resulted in significantly lower levels of intracellular QD staining. Flow cytometry measurements showed that under these modified conditions approx. 20% of the cells are labelled with the nanohybrids. In comparison, no signal was measured from cells incubated with nanohybrids prepared in the absence of His_6_-MBP-γ (Figure S5 in Supporting Information File 1).

**Figure 2:**
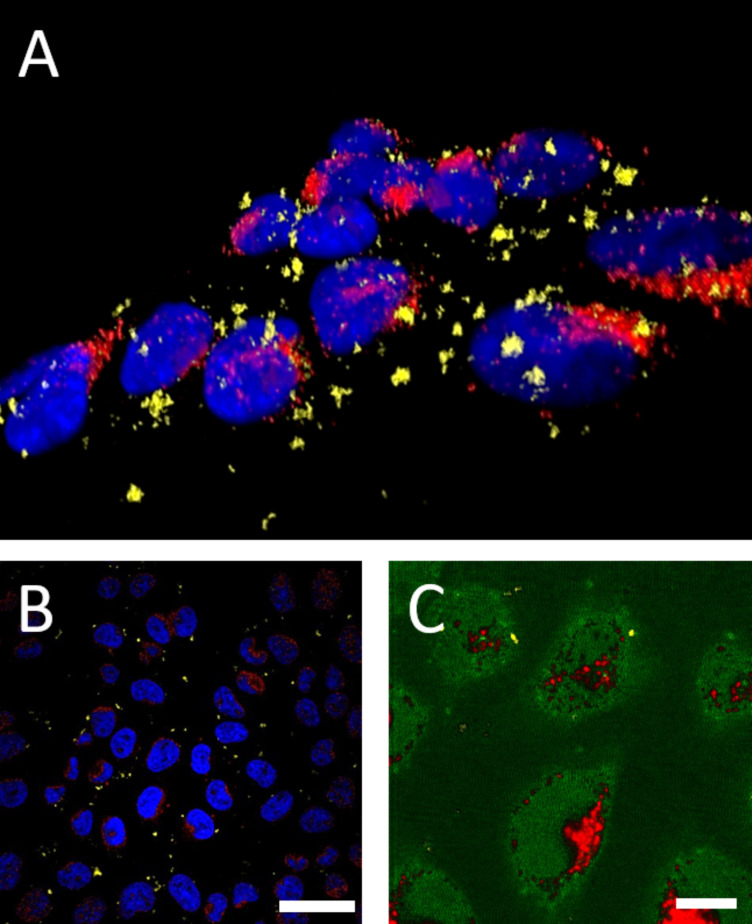
Confocal z-stack image of HeLa cells. (A) Volume view of the confocal z-stack showing blue: DAPI, red: endosomal marker Cy5, yellow: QD signal. (B) Spectral scan of one confocal plane (same colour code, scale bar 50 µm) (C) spectral unmixing of QD fluorescence (575 nm) and Cy5-spectrum (red) highlighting no superposition between the QD and the Cy5 signal, scale bar 10 µm.

To gain further insight into the distribution of QD stain, we visualized the cell cultures incubated with a lower nanohybrid concentration and lower MBP-γ loading of per nano-assembly, using confocal microscopy (Figure 1E). A close examination of the images allows us to distinguish three different colour distributions: the cell nuclei shown in blue (stained with DAPI), the endosomal compartments counterstained in red (labelled with Cy5-transferin), and QDs in yellow. The images clearly indicate that QDs and Cy5-transferrin do not co-localize. In addition, the dark signals observed in bright field mode, coincide with the yellow fluorescence emitted when we switch to fluorescence mode. This indicates that these spot signals are assemblies of multiple hybrid particles, containing AuNPs and encapsulated QDs [3]. Similar features were reported in a recent publication of the Jana group [4]. Due to the colloidal stability of these constructs as verified by DLS, we assume that the appearance of these rather large structures/patterns is due to the *cellular fate* of these structures rather than appearance in solution. The confocal microscopy data were further exploited to generate a z-stack, to visualize the fluorescence distribution of the nanocomposites side-by-side with that of the Cy5 dye and cell nuclei. The 3D-stack, shown in Figure 2A, confirms that distribution of the internalized nanohybrids (yellow staining) is distinct from that of the endosomes (counterstained in red). This provides further confirmation of the data shown in Figure 1E, demonstrating that the nanohybrids are not trapped within endocytic vesicles.

The respective signals were further identified using spectral resolution of the emission associated with the three chromophores. The spectral scan of one confocal plane (in Figure 2B) shows different locations for the nanohybrids (yellow), endosomal marker (red), and cell nuclei (blue). Spectral unmixing was also applied to a region, where the QD fluorescence staining is close to the Cy5-transferrin associated with the endosomal marker (Figure 2C). The two stainings corresponding to the nanohybrids and Cy-5-transferrin do not share the same compartments. Clearly, these findings combined show that the nanocomposites, when internalized, are found in subcellular compartments that are distinct from those stained with the Cy5-transferrin. These results are in good agreement with our previous findings reported in reference [22]. These results suggest that the mechanism of cellular uptake promoted by the gamma peptide may not be driven by endocytosis [22]. Nonetheless, the distribution of the QD fluorescence is still different from that expected for a pure cytosolic delivery, where a more homogeneous distribution of the signal would be expected [33]. Whether these findings are due to the cellular response on the NP-based structure of the hybrid, or they reflect the typical cellular fate of a non-enveloped virus is a question that cannot be easily addressed. However, it is worth noting that even a small amount of γ-peptide (an average of ≈7 γ-peptide per nanohybrid) can promote the uptake of nanohybrids, which hydrodynamic size exceeds both the QD construct of our previous study (d(QD-LA) ≈ 10–15 nm [22]) or the virus particle itself (*d* ≈ 40 nm [21]).

## Experimental

In brief, the functionalized polymer-encapsulated quantum dots were left to incubate with partially capped gold nanoparticles. This conjugate was subsequently functionalized with His_6_-MBP-γ using self-assembly processes. For controls His_6_-MBP was used. The processes are described in more detail in Supporting Information File 1. In reference [8] further details on the hybrid characterization can be found.

## Supporting Information

Expression of the fusion protein His_6_-MBP-gamma, particle synthesize, hybrid assembly and characterization, DLS characterization and colloidal stability assessment, cellular incubation, amylose column, HeLa cellular culture, epifluorescence z-stack, epifluorescence control experiments, flow cytometry, further instrumentation.

File 1 Additional experimental data.
