# Supplementary material for: Small protein sequences can induce cellular uptake of complex nanohybrids
Source: Beilstein J Nanotechnol. 2019 Dec 12;10:2477–82. doi: 10.3762/bjnano.10.238 (PMC6941447; doi:10.3762/bjnano.10.238)
Supplement: File 1 — Additional experimental data. [file Beilstein_J_Nanotechnol-10-2477-s001.pdf]

BEILSTEIN JOURNAL OF NANOTECHNOLOGY

Supporting Information

for

Small protein sequences can induce cellular uptake of complex nanohybrids

Jan-Philip Merkl, Malak Safi, Christian Schmidtke, Fadi Aldeek, Johannes Ostermann, Tatiana Domitrovic, Sebastian Gärtner, John E. Johnson, Horst Weller and Hedi Mattoussi

Beilstein J. Nanotechnol. **2019,** *10,* 2477–2482. doi:10.3762/bjnano.10.238

Additional experimental data

License and Terms: This is a supporting information file under the terms of the Creative Commons Attribution License (https://creativecommons.org/licenses/by/4.0). Please note that the reuse, redistribution and reproduction in particular requires that the authors and source are credited.
The license is subject to the *Beilstein Journal of Nanotechnology* terms and conditions: (https://www.beilstein-journals.org/bjnano)

Table of contents

Self-Assembly and characterization of the nanohybrids S3
DLS characterization S3
Amylose affinity chromatography assay S4
Cell culture preparation and incubation protocol S4
Epifluorescence microscopy S5
Control experiments... S6
Flow cytometry.. S7
Recerences S8

Self-Assembly and characterization of the nanohybrids

Growth of the quantum dots (QDs),[1,2] growth of the zwitterion-capped gold nanoparticles (LA-ZW-AuNPs),[3,4] synthesis of the polymers[2,5,6], and the nanohybrid assemblies[7] have been discussed in details in our previous works.[2-8] Briefly, QDs synthesized in high boiling solvents[1,2] were transferred into water using ligand exchange with polyisoprene-diethylentriamine (PI-DETA) followed by encapsulation within a functional amphiphilic polyisoprene-*block*-polyethylene oxide diblock copolymer (PI-*b*-PEO-FG, where FG = lipoic acid, amine, carboxylic acid). [2,5,6,7] The AuNPs were grown in the presence of lipoic zwitterion ligands.[3,4] The hybrid design is similar to the one reported in reference,[7] except the His-MBP-γ peptide used here allows testing of intracellular uptake. Different PI-*b*-PEO-FG coatings were prepared and tested. However, the results presented in this report focused on the use of PI-*b*-PEO-NH_2 copolymer.

To investigate the biological activity of the monohybrids, different stoichiometry of the AuNPs and QDs were used. In a typical preparation, dispersions of QD-FG and AuNPs in PBS buffer (20 mM, pH 7.2) were mixed and incubated for 30 min at room temperature in the dark. Then, a solution of HIS-MBP-γ (at 14 equiv per AuNP, with a final QD concentration of ≈1 µM) for 30 min. For the cell incubation, the stock solution was diluted in cellular DMEM culture media (final volume 500 µL) prior to use. The concentration of the nanohybrids usually refers to the QD-concentration, unless otherwise stated.

DLS characterization

The hydrodynamic diameter of the nanoparticles or nanohybrids were measured using an ALV/CGS-3 Compact Goniometer System equipped with an avalanche photodiode for signal detection and ALV photon correlator (Langen, Germany). The signal was collected at 90° scattering angle. Each scattered pattern was the average of 3 acquisition periods of 10 seconds each. The intensity count rates were maintained at ≈150–300 Hz, achieved through appropriate control over the NPs or the nanohybrid concentrations used. The resulting autocorrelation function was fitted to a cumulant series using ALV-7004 software. The Laplace transform was then applied to obtain an intensity distribution function versus the hydrodynamic diameter. The hydrodynamic diameter D_H measured for the nanohybrids is ≈40–50 nm (intensity average) is approximately the sum of D_H(QD-NH_2) ≈30 nm, D_H(AuNPs) ≈10 nm, and the size of His-MBP (≈3 nm). We propose assume that a nanohybrid is typically made of a central QD, surrounded by few quantum AuNPs, and each AuNPs is coupled to a few MBPs. The nanohybrids were colloidal stable over several weeks in buffer media, and for at least 5 h when mixed with growth media (duration of the tests).

We also collected the autocorrelation function of the hybrids before and after incubation with the cell culture media. No difference was found on time scales, as shown in Figure S1. This indicates absence of aggregate formation of the hybrid materials under the conditions explored.[8]

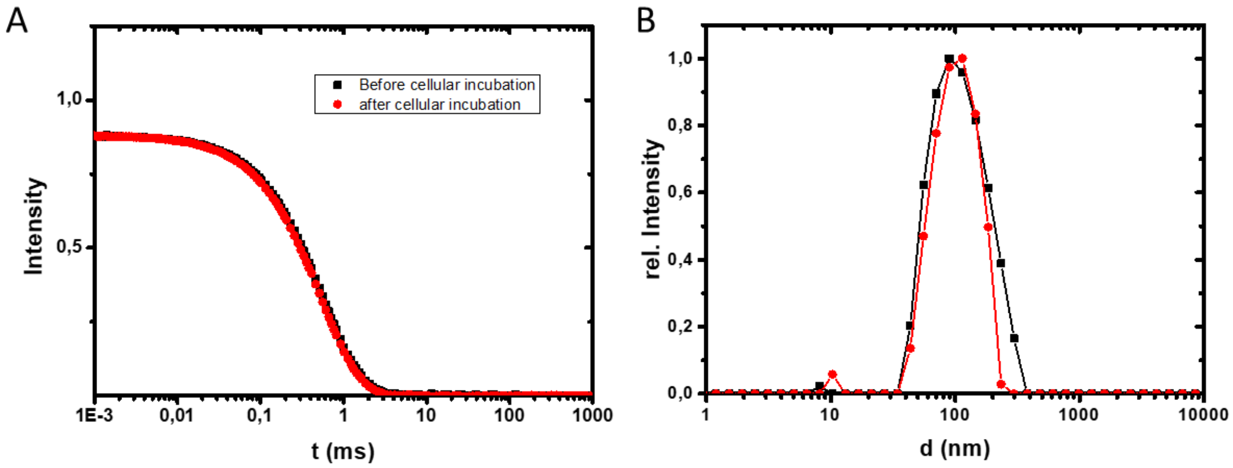

Figure S1: A: Intensity autocorrelation function acquired from the nanohybrids before (black) and after incubation with the cell culture media (red) for ~1 h, B: intensity distribution extracted from A.

Amylose affinity chromatography assay

Expression of the His_7-MBP-gamma-peptide (MBP-γ) fusion was detailed in reference.[9] Binding of the fluorescent-plasmonic assembly to the amylose column relied on a visual inspection. Briefly, 1.5–2 mL of amylose stock gel was loaded onto a 10 mL capacity column, and washed several times with 10 mL of PBS buffer (20 mM, pH 7.2). The nanohybrids dispersion (≈100 nM of QDs in PBS buffer) were first loaded onto the column, yielding a diffuse band on top of the column that looks yellowish under UV irradiation (from the QD fluorescence) and purple under white light (from the AuNP SPR). The column was washed several times with buffer solution with no effects on the immobilized band, confirming that tight binding to amylose has taken place. Elution of the hybrids was achieved by adding a few mL of *D*-maltose solution (20 mM).[3]

Cell culture preparation and incubation protocol

HeLa cell lines were provided by the Florida State University (Tallahasse, FL, USA) cell culture facility. The cells were cultured in complete growth medium (Dulbecco's Modified Eagle's 15 Medium, DMEM, Corning Cellgro) supplemented with 4.5 g/L glucose, *L*-glutamine, sodium pyruvate, 1% (v/v) antibiotic-antimycotic 100x (Gibco), 1% (v/v) non-essential amino-acid solution 100x (Sigma), and 10% (v/v) fetal bovine serum (FBS, from Gibco). The cells were grown as a monolayer in a T25-flask at 37 °C under a humidified 5% CO_2 atmosphere. A subculture was performed every 2-4 days.

HeLa cells (≈8 × 10^4) were seeded onto 18 mm circle micro-cover glasses placed into 24-well microtiter plates (CellStar, VWR), and the plates were placed in an incubator (37 °C, humidified 5% CO_2 atmosphere) overnight to allow attachment and recovery. After 24 h, the different nanocomposites, diluted into culture medium (DMEM without phenol red, Invitrogen), were added. The cultures were incubated at 37 °C for 1 h, and then Cy5-transferrin marker (at 40 µg/mL) was added to the culture

to identify the late endosomal/lysosomal compartments.[9] Excess of unbound QD reagents and Cy5 were removed by washing three times with phosphate-buffered saline (PBS, pH 7.4). The cells were fixed with paraformaldehyde (3% solution, 12 min at room temperature, washed and mounted in ProLong Antifade mounting media containing DAPI dye (Invitrogen) for nuclear staining, and then imaged.

Epifluorescence microscopy

Epifluorescence images of the fixed cells were collected using a Nikon Ti-E inverted microscope, equipped with an Intensilight C-HGFI illuminator and a Photometrics Cool Snap HQ2 camera (Photometrics). Images were collected using a 40x objective (Nikon) and a set of filter cubes purchased from Chroma Technology (Rockingham, VT). Excitation of all samples was provided by a Xe lamp. DAPI fluorescence was detected using a DAPI cube (D350/50x for excitation, dichroic 400DCLP, and D460/50m for detection). The QD fluorescence signal (peak at 575 nm) was detected using a GFP/EGFP cube (HQ470/40x for excitation, dichroic Q495lp, and HQ525/50m for detection), while the Cy5 emission was detected using Tx-Red cube (HQ560/55x for excitation, dichroic Q595lp, and HQ645/75 for detection). The images were analyzed and processed using NIS-Elements Advanced 16 Research (Nikon) and Photoshop Adobe (software). Differential interference contrast (DIC) images were collected using a bright light source. An epifluorescence z-stack was applied to the HeLa sample, where very high cellular uptake was measured (Figure S2 from two different perspectives).

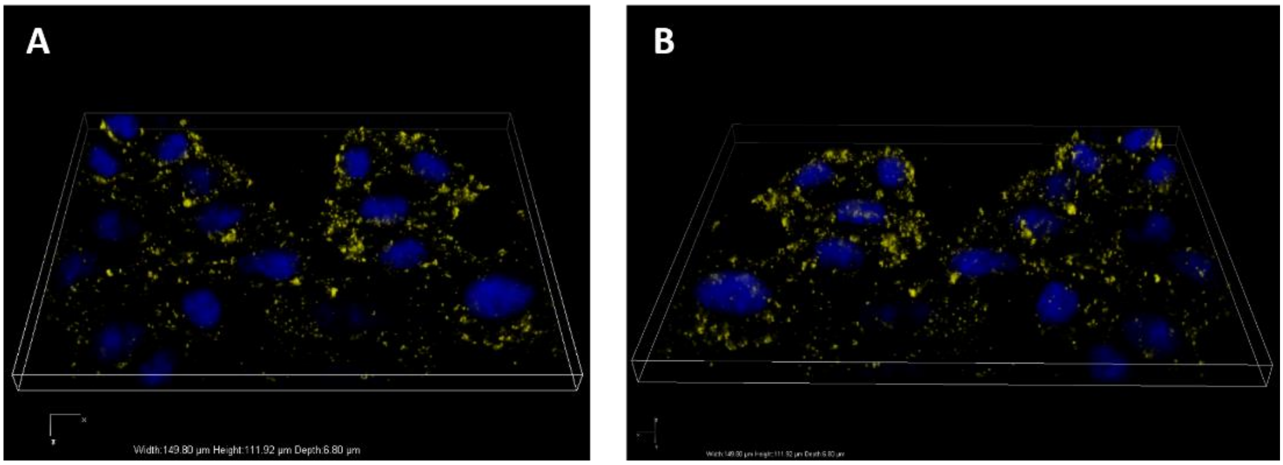

Figure S2: Epifluorescence z-stack acquired from HeLa cells after incubation with the nanohybrids for of 1 h (nanohybrid concentration ≈100 nM, 14 equiv His-MBP-γ per AuNP were used). No co-incubation with Cy5-transferrin was included. The panels A and B show the same picture viewed from two different perspectives. The displayed volume is 150 × 111 × 6.8 μm^3.

Control experiments

Control experiments were carried out to confirm that the presence of His-MBP-γ bound to the AuNPs in the nanohybrids is required for promoting interactions with the cells. For this, HeLa cells were incubated with different reagents combinations then imaged. First, a z-stack fluorescence image collected from cells incubated with the nanohybrids containing His-MBP (no γ peptide) for 1 h is shown in Figure S3, after washing with buffer. No uptake is measured. Second, the fluorescence images in Figure S4 were collected from HeLa cell culture incubated alone, HeLa cell culture incubated with QD-NH_2, and HeLa cell culture incubated with a mixture of QD-NH_2 and His-MBP-γ (no AuNPs). No cellular uptake is observed in any of the images shown, which indicates that the presence of AuNP-bound His-MBP-γ in the nanohybrid is required. The data in Figure S4 also indicate that His-MBP-γ does not interact with the fully shielded encapsulated-QDs, as previously discussed.[2]

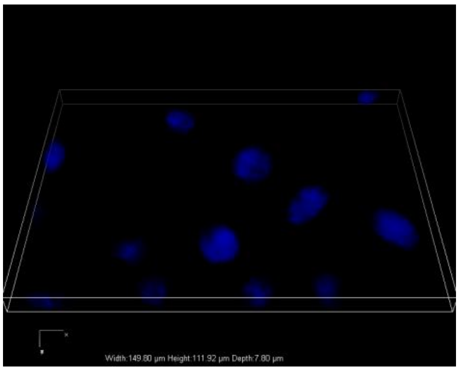

Figure S3: Epifluorescence z-stack acquired from a control cultures made of HeLa cells incubated with nanohybrids containing His-MBP (no γ peptide) for 1 hour after washing with buffer. The displayed volume is 150 × 111 × 7.8 µm^3.

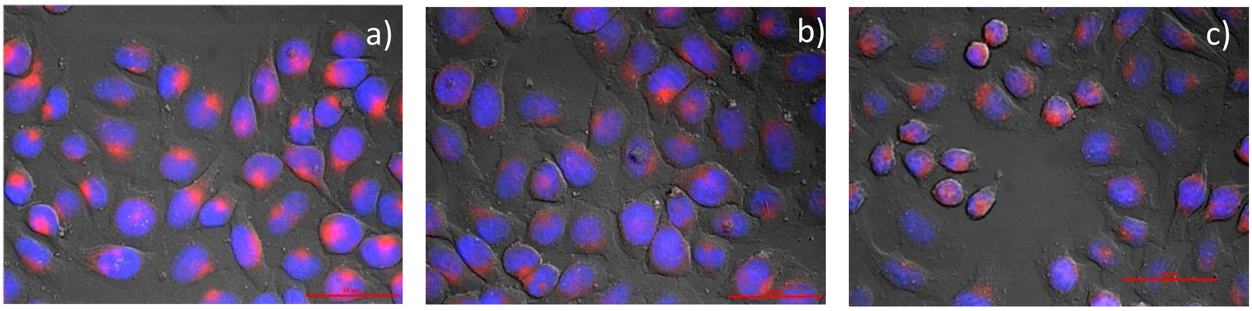

Figure S4: Investigation of the cellular uptake by HeLa cells: *c*(QD) = 100 nM, incubation time = 1 h. Merged image of DIC, DAPI channel (blue), Transferin-Cy5 Channel (red) and QD channel (yellow). Shown are HeLa cells (a) incubated with the cell culture (control), (b) incubated with QD-NH_2, and (c) with QD-NH_2 mixed with 12 equiv of His-MBP-γ (i.e., not attached via AuNPs). Cells were also incubated with Cy5-transferrin.

Flow cytometry

Cells were first treated with different concentrations of the nanohybrids presenting bound His-MBP-γ for 1 h; 500 mL aliquot of the nanohybrid dispersion was used in each experiment. For control, nanohybrids presenting bound His-MBP (no γ) were used. After incubation, the cells were washed with PBS buffer three times. After trypsinization, cells were centrifuged at 1200 rpm for 5 min, resuspended in cell culture medium, and then analyzed by flow cytometry. Forward scatter (FS), side scatters (SS), and fluorescence of individual cells were measured on a BD FACS Aria (SORP) flow cytometer. Forward scatter FS and SS intensities (measured at 488 nm) were digitalized on both linear and logarithmic scale (4 decades). No propidium iodide was used. Dead cells and living cells were identified by their forward scatter. Samples were excited with a 561 nm laser and emission was collected using a 585/40 bandpass emission filter. These experiments were performed under "lower uptake conditions" meaning a reduced QD to AuNP ratio of 1:1 and replacement of 50% of the His_7-MBP-γ with unreactive His_7-MBP. The flow cytometry data confirms that the cellular uptake occurs only in the presence of the lytic γ-peptide.

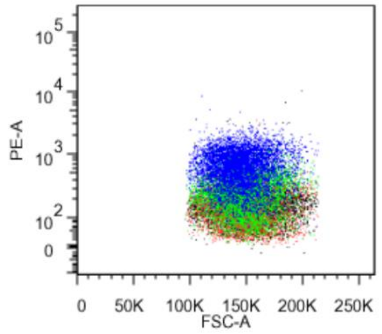

Figure S5: Flow cytometry data. Forward scatter against 585/40 bandpass emission filter after a cellular incubation of 1 h. (Black) HeLa cells, control. (Red) Nanohybrids incubated with HIS_7-MBP, (green and red) nanohybrids carrying His_7-MBP-γ, with green corresponding to c(nanohybrids) ≈ 50 nM and blue corresponding to c(nanohybrids) ≈100 nM.

REFERENCES

(1) Talapin, D. V.; Mekis, I.; Götzinger, S.; Kornowski, A.; Benson, O.; Weller, H. CdSe/CdS/ZnS and CdSe/ZnSe/ZnS Core-Shell-Shell Nanocrystals. *J. Phys. Chem. B* **2004**, *108*, 18826–18831.

(2) Ostermann, J.; Merkl, J.-P.; Flessau, S.; Wolter, C.; Kornowksi, A.; Schmidtke, C.; Pietsch, A.; Kloust, H.; Feld, A.; Weller, H. Controlling the Physical and Biological Properties of Highly Fluorescent Aqueous Quantum Dots Using Block Copolymers of Different Size and Shape. *ACS Nano* **2013**, *7*, 9156–9167.

(3) Aldeek, F.; Safi, M.; Zhan, N.; Palui, G.; Mattoussi, H. Understanding the Self-Assembly of Proteins onto Gold Nanoparticles and Quantum Dots Driven by Metal-Histidine Coordination. *ACS Nano* **2013**, *7*, 10197–10210.

(4) Aldeek, F.; Muhammed, M. A. H.; Palui, G.; Zhan, N.; Mattoussi, H. Growth of Highly Fluorescent Polyethylene Glycol- and Zwitterion-Functionalized Gold Nanoclusters. *ACS Nano* **2013**, *7*, 2509–2521.

(5) Pöselt, E.; Schmidtke, C.; Fischer, S.; Peldschus, K.; Salamon, J.; Kloust, H.; Tran, H.; Pietsch, A.; Heine, M.; Adam, G.; *et al.* Tailor-Made Quantum Dot and Iron Oxide Based Contrast Agents for in Vitro and in Vivo Tumor Imaging. *ACS Nano* **2012**, *6*, 3346–3355.

(6) Schmidtke, C.; Pöselt, E.; Ostermann, J.; Pietsch, A.; Kloust, H.; Tran, H.; Schotten, T.; Bastús, N. G.; Eggers, R.; Weller, H. Amphiphilic, Cross-Linkable Diblock Copolymers for Multifunctionalized Nanoparticles as Biological Probes. *Nanoscale* **2013**, *5*, 7433–7444.

(7) Merkl, J.-P.; Schmidtke, C.; Aldeek, F.; Safi, M.; Feld, A.; Kloust, H.; Mattoussi, H.; Lange, H.; Weller, H. Functional-Group-Dependent Formation of Bioactive Fluorescent-Plasmonic Nanohybrids. *J. Phys. Chem. C* **2016**, *120*, 25732–25741.

(8) Albanese, A.; Walkey, C. D.; Olsen, J. B.; Guo, H.; Emili, A.; Chan, W. C. W. Secreted Biomolecules Alter the Biological Identity and Cellular Interactions of Nanoparticles. *ACS Nano* **2014**, *8*, 5515–5526.

(9) Safi, M.; Domitrovic, T.; Kapur, A.; Zhan, N.; Aldeek, F.; Johnson, J. E.; Mattoussi, H. Intracellular Delivery of Luminescent Quantum Dots Mediated by a Virus-Derived Lytic Peptide. *Bioconjug. Chem.* **2017**, *28*, 64–74.
